# Human-in-the-Loop Enhances Machine Learning Inference in Intraoperative Optical Coherence Tomography Glioma Imaging

**DOI:** 10.3390/medsci14020263

**Published:** 2026-05-20

**Authors:** Radik Zinatullin, Alexander Sovetsky, Artem Grishin, Elena Kiseleva, Liudmila Kukhnina, Svetlana Korikova, Alexander Matveyev, Vladimir Zaitsev, Konstantin Yashin, Lev Matveev

**Affiliations:** 1 Russian Academy of Sciences, Institute of Applied Physics, 46 Ulyanov Str., 603951 Nizhny Novgorod, Russia; neuradik@yandex.ru (R.Z.); alex.sovetsky@ipfran.ru (A.S.); matveyev@ipfran.ru (A.M.); vyuzai@mail.ru (V.Z.); jashinmed@gmail.com (K.Y.); 2 Department of Neurosurgery, Privolzhsky Research Medical University, 10/1, Minin and Pozharsky Sq., 603950 Nizhny Novgorod, Russia; zhest8242@mail.ru (A.G.); kiseleva84@gmail.com (E.K.); kuhnina.ludmila@yandex.ru (L.K.); korikovaa_sveta@mail.ru (S.K.)

**Keywords:** human-in-the-loop, optical coherence tomography, glioma, machine learning, intraoperative imaging, optical biopsy

## Abstract

**Background/Objectives**: The integration of Artificial Intelligence (AI) into clinical workflows raises critical questions regarding decision-making responsibility, as fully autonomous systems inevitably carry a margin of error that can be fatal in high-stakes fields like surgery. This study addresses this challenge by evaluating a “Human-in-the-Loop” (HITL) workflow, using intraoperative Optical Coherence Tomography (OCT) for glioma detection. We aimed to determine if integrating Machine Learning (ML)-generated segmentation maps with human contextual analysis resolves the tension between automation and clinical responsibility, yielding superior diagnostic reliability compared to structural or quantitative imaging alone. **Methods**: We retrospectively analyzed 86 intraoperative OCT scans from 27 patients. Five neurosurgeons blindly assessed the data across three progressive levels of processing: (1) structural scans, (2) physics-based parametric maps, and (3) SVM-based generated segmentation maps. Crucially, the HITL inference performance on segmentation maps was benchmarked against “models-only” inference pipeline: a SVM and a state-of-the-art multimodal reasoning model, Gemini 3.1 Pro. To evaluate interpretability and the operator’s ability to confidently exercise their authority, we measured inter-rater consistency alongside diagnostic performance. **Results**: The results demonstrate that, while quantitative parametric maps improved Global Accuracy (87% [95% CI: 82–92%]) compared to structural scans (80% [95% CI: 73–86%]), they suffered from an “interpretability gap,” resulting in a moderate inter-rater consistency of 0.68 [95% CI: 0.59–0.78]. In contrast, the HITL approach using segmentation maps maximized consensus to 0.98 [95% CI: 0.95–1.00] and achieved the highest performance (Accuracy 94% [95% CI: 88–98%] and Sensitivity 98% [95% CI: 92–100%]). Compared to the standalone models, the HITL approach significantly outperformed the SVM baseline (Accuracy 84% [95% CI: 81–87%]; Sensitivity 83% [95% CI: 78–88%]). Furthermore, it surpassed the SOTA Gemini 3.1 Pro model (Accuracy 90% [95% CI: 83–95%]; Sensitivity 86% [95% CI: 74–95%]). While the HITL sensitivity demonstrated a definitive and statistically significant edge over the Gemini model, the accuracy improvement fell just slightly short of undisputed statistical significance due to overlapping confidence intervals. **Conclusions**: By utilizing their clinical domain knowledge of tumor invasion patterns and topological priors, surgeons effectively filtered algorithmic noise—overriding ML errors in 69% (9 out of 13) false positive cases that models alone could not resolve. This demonstrates exactly how and where HITL optimally utilizes human contextual intelligence to outperform autonomous “models-only” pipelines, confirming a human-ML synergy that augments the objectivity of machine learning with human domain knowledge. This paradigm ensures that the ultimate responsibility for diagnostic inference remains safely and practically in human hands. **Open Data Initiative**: To ensure essential reproducibility, enable independent multi-center validation and support open science, all examples of intraoperative in vivo OCT brain scans used in this study are made publicly available. To the best of our knowledge, this represents the first open-access data of its kind globally.

## 1. Introduction

The rapid advancement of Artificial Intelligence (AI) in healthcare has brought unprecedented computational capabilities to clinical diagnostics. However, the transition toward fully autonomous decision-making remains fundamentally constrained by the inherent limitations of predictive models. Even when a highly optimized AI system achieves a 90% accuracy rate, the remaining 10% of false predictions represents a critical vulnerability. In high-stakes environments such as surgery, these unavoidable edge cases and algorithmic hallucinations can lead to fatal consequences. Consequently, a paradigm shift is occurring: rather than striving for complete automation, the focus is increasingly placed on designing systems where a human operator acts as an essential safety net, augmenting the AI through deep domain knowledge to identify and filter out errors that the model itself cannot resolve.

This shift is not merely a technical necessity but a fundamental ethical requirement to address the “diffusion of responsibility” in AI-assisted decision-making. When an autonomous model makes an error, attributing blame solely to the algorithm or its developers is legally and clinically untenable. In medical practice, the ultimate responsibility and liability always remain with the human clinician [1]. Therefore, transferring control to an AI system without providing the human operator with the means to effectively supervise, interpret, and overrule the model creates an unacceptable risk. To ensure safe implementation, Human-Centered AI (HCAI) and “Human-in-the-Loop” (HITL) architectures are required. These frameworks pivot the dynamic from machine autonomy to human–machine collaboration, ensuring that the human remains at the locus of control and responsibility [2,3].

It is important to clarify that the concept of “Human-in-the-Loop” encompasses a broad spectrum of human–machine interactions across different phases of the AI lifecycle. Human oversight, feedback, and decision-making can be integrated at various stages of the AI pipeline, most notably during the active model training (learning phase) and the operational application (inference phase). To accurately reflect our contribution, we emphasize that our study is exclusively devoted to a pure HITL approach at the inference stage. Rather than presenting a human-in-the-loop learning framework where human feedback continuously retrains the algorithm, our work focuses on improved human-assisted interpretation. We demonstrate how human contextual intelligence enhances the final diagnostic decision-making based on static machine learning outputs [4].

Within a HITL framework, the human operator does not passively accept AI outputs; rather, they actively contextualize them. Effective human–AI collaboration requires the model to present its inferences in a highly explainable manner, allowing the clinician to apply their domain knowledge—such as anatomical constraints, physiological plausibility, and topological priors—to validate or reject the AI’s suggestions [5]. By clearly defining decision-making roles, the human operator can compensate for the AI’s lack of contextual awareness, while the AI compensates for human cognitive fatigue or subjective bias [6].

This broad paradigm of human-centered inference and responsibility is acutely relevant in the specific context of neurooncology. Malignant gliomas are the most common primary brain tumors, and the extent of surgical resection is directly related to patient life expectancy [7]. However, tumor resection under conventional white light microscopy frequently fails to achieve a total resection, as diffusely infiltrating tumor cells are visually indistinguishable from healthy white matter [8]. Consequently, there is a critical need for technologies that can enhance the accuracy of determining the resection area.

The most popular current methods are intraoperative magnetic resonance imaging (iMRI), intraoperative ultrasound (iUS), and 5-aminolevulinic acid (5-ALA) fluorescence-guided surgery [9,10]. These methods provide real-time data and are not compromised by brain shift. Nonetheless, they possess significant drawbacks, including high cost (iMRI), low spatial resolution (iUS), and the need for contrast agents coupled with a dependence on tumor grade (5-ALA). Moreover, access to these technologies is often limited in operating rooms across various clinics [11,12,13,14]. Under these conditions, optical biopsy techniques hold significant potential.

Methods such as Optical Coherence Tomography (OCT), Fluorescence Lifetime Imaging (FLIM), and Raman spectroscopy are being actively investigated. The high resolution of OCT makes it particularly suitable for delineating the borders of the peritumoral zone. This capability allows for a more complete resection of affected tissue [15]. OCT exhibits great potential for clinical translation, thanks to the availability of certified systems for intraoperative use. The working principle of OCT is similar to ultrasound B-mode imaging but utilizes reflected near-infrared light rather than acoustic waves [16].

It is well-established that the optical properties of tumor tissue and intact white matter differ. These differences manifest in visual criteria on OCT scans, such as signal attenuation depth, the uniformity of signal attenuation, signal homogeneity, and the presence of a detectable signal in the orthogonal polarization channel [17]. Quantitative optical parameters, including the attenuation coefficient, speckle contrast, and depolarization ratio, also exhibit significant differences [17,18].

However, the direct clinical application of these optical contrasts faces exactly the challenges of interpretation and responsibility outlined above. The diagnostic accuracy of qualitative structural analysis is often insufficient for reliable decision-making due to inherent subjectivity and the masking of useful information by confounding factors (such as attenuation artifacts and speckle noise) [18]. Similarly, while quantitative parametric maps mitigate some of these issues by visualizing direct informational patterns, they present ambiguous, continuous variables that are cognitively demanding to interpret. Conversely, fully automated ML-segmentation inevitably yields a certain percentage of classification errors at the individual A-scan level (e.g., false positives). Fully formalizing the complex biological context—such as the probability of specific tissue combinations and invasion patterns—into an autonomous model is computationally challenging.

Therefore, intraoperative OCT provides an ideal case study for examining the efficacy of HITL systems. Based on the necessity of maintaining clinical responsibility while maximizing accuracy, this study aims to evaluate the diagnostic performance and interpretability of a HITL approach across three distinct levels of data processing: qualitative assessment of structural OCT scans, analysis of quantitative optical parameter maps, and interpretation of SVM-generated segmentation maps. Crucially, to explicitly quantify the diagnostic advantage of human contextual intelligence over autonomous algorithms, this study further aims to benchmark the HITL inference directly against “models-only” pipelines, specifically evaluating it alongside the standalone SVM classifier and a state-of-the-art multimodal reasoning Vision-Language Model (VLM), Gemini 3.1 Pro. To ensure a rigorous comparison, the Gemini model evaluated the SVM-classification-based segmentation maps utilizing few-shot in-context learning. This evaluation was guided by a structured text prompt providing a description of the visual heuristics associated with cancer cell infiltration, alongside an explicit caution to the model regarding the probability of false-positive errors within the segmentation map patches.

## 2. Materials and Methods

### 2.1. OCT Setup

Structural scans were acquired in vivo using a time-domain polarization-sensitive optical coherence tomography system (OCT-1300U, BioMedTech LLC, Nizhny Novgorod, Russia) in the operating room. The scanner specifications were as follows: central wavelength 1300 nm, B-scans covers laterally 2 mm with 256 pixels in depth and 512 pixels laterally. Axial pixel size is 7.5 μm and lateral is 4 μm (with lateral FWHM beam size of 25 μm).

Each volumetric scan (2 mm × 2 mm field of view) is captured in 3 s. The current implementation of the segmentation process takes approximately 2 min (including transferring the data from the OCT device to the further processing software described in subsections below). This represents a significant time reduction compared to traditional rapid-frozen section biopsies, which require 30–40 min and offer absolutely no alternative for in vivo cases.

### 2.2. Population and Inclusion Criteria

The study included all patients with histologically confirmed malignant brain gliomas. The collected dataset is uniquely valuable: all images were acquired directly during intraoperative in vivo OCT imaging, followed by the precise excision of the region of interest for subsequent pathomorphological examination. We included scans with definitively confirmed tumor or definitively confirmed white matter. The study cohort consisted of 27 patients (mean age 52 years) who underwent a total of 86 scans. The sample included 44 scans of white matter and 42 tumor scans. The tumor scans comprised the following histologies: grade I glioma (n = 1), grade II glioma (n = 2), grade III glioma (n = 3), grade IV IDH-mutant astrocytoma (n = 6), and glioblastoma (n = 9).

### 2.3. OCT Scans Preprocessing and Parametric Maps

**Figure 1:**
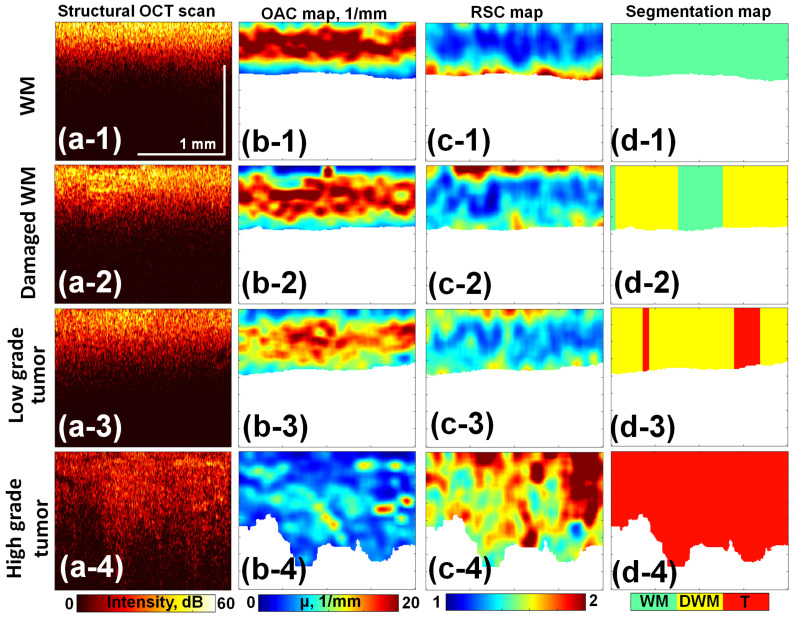
Columns display the progressive stages of data processing: (**a**) Structural OCT scans. (**b**) Optical Attenuation Coefficient (OAC) maps. (**c**) Refined Speckle Contrast (RSC) maps. (**d**) Final segmentation maps. Rows illustrate the representative tissue classes: (**1**) White Matter (WM). (**2**) Damaged WM. (**3**) Low-grade tumor. (**4**) High-grade tumor.

In this study, we utilized the following: structural intraoperative OCT scans (Figure 1a), OCT scans processed by OAC and Refined speckle contrast (RSC) (Figure 1b,c), and segmentation maps (Figure 1d), generated using a 2D-parametric classifier. The preprocessing of B-scans to generate OAC and RSC parametric maps was performed using the approach described in [18]. Briefly, the optical attenuation coefficient (OAC) is calculated per A-scan as follows:

(1) $$\mu_{s}\left(j\right)=\frac{I(j)}{2\Delta \sum_{i=j}^{\infty }I\left(i\right)}$$

where I(j) is the intensity of OCT signal in the *j*-th pixel in an A-scan, and Δ is the axial inter-pixel distance to enable the correct dimensionality of the depth-resolved attenuation coefficient $\mu_{s}$. We assume here that in the pixel with the maximal index *∞*, intensity *I* tends to zero. The RSC parameter is calculated using the OAC to compensate for depth-dependent signal decay, ensuring that it reflects true microstructural inhomogeneities rather than attenuation artifacts [18]:

(2) $$RSC=\frac{\sqrt{\langle \mu_{s}^{2}\rangle -{\langle \mu_{s}\rangle }^{2}}}{\langle \mu_{s}\rangle }$$

To calculate the RSC while ensuring appropriate speckle statistics in B-scan, a 10 × 20 px (depth × width) sliding window is applied.

To prevent artifacts resulting from a low signal-to-noise ratio (SNR) at depth in structural images, a signal intensity cut-off threshold for OAC and RSC maps is set at the noise-floor (15 dB). The OAC and RSC values were matched with tumor and white matter regions defined based on histological results. A Support Vector Machine (SVM) model was trained, and segmentation maps were generated as the classifier output. These maps visualize the classification of A-scans into specific tissue types (tumor, damaged white matter, or normal white matter) based on their parametric values.

### 2.4. SVM-Classification-Based Segmentation

To automate the generation of segmentation maps presented to the surgeons, we employed an SVM classifier with a linear kernel [19]. The classification was based on a two-dimensional feature space comprising OAC and RSC [18]. For each B-scan segment (comprising 20 adjacent A-scans), the mean values of OAC and RSC were calculated within a specific region of interest (depth 20–40 pixels) to form the feature vector. Compared to our previous work [18], where parameters were averaged across the entire B-scan width (400 A-scans), reducing the analysis window to 20 A-scans allowed us to account for the inherent performance drop associated with smaller analytical windows, even on the same homogeneous training dataset. Importantly, when evaluating heterogeneous scans to detect cancer cell invasion, this localized windowing is a critical step for generating high-resolution spatial maps of different tissue types. Based on this mapping, surgeons engage in HITL inference by applying their clinical understanding of tumor invasion patterns. They perceptually contrast these biologically plausible patterns against the algorithm’s potential error probabilities—which inevitably arise from the small window size—making this contextual evaluation a vital element of human inclusion specifically at the inference stage.

**Figure 2:**
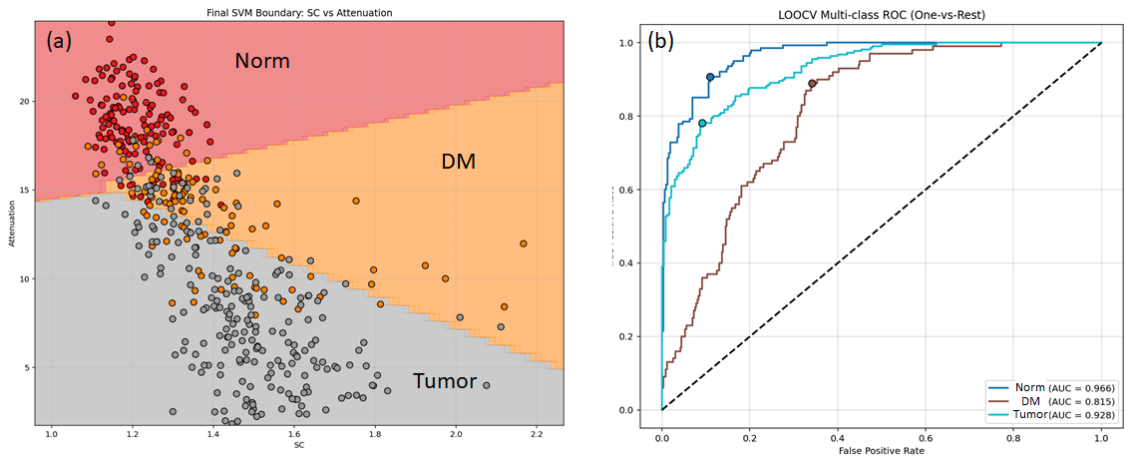
Automated SVM-based classification of OCT scans. (**a**) Decision boundaries of the linear SVM classifier trained on the two-dimensional feature space (Speckle Contrast and Attenuation) for Normal tissue (Norm), Damaged Matter (DM), and Tumor. (**b**) Leave-One-Out Cross-Validation (LOOCV) Multi-class Receiver Operating Characteristic (ROC) curves, demonstrating high Area Under the Curve (AUC) values for all tissue types, with the target Tumor class achieving an AUC of 0.928. The black dashed line on (**b**) indicates the performance of a random classifier (AUC = 0.5).

To prevent overfitting, the SVM model was trained on 23 B-scans from a previous study [18]. Crucially, these training scans were selected because they were strictly homogeneous, with their respective tissue states reliably confirmed by matched histology. Specifically, each of the 23 B-scans, consisting of 400 A-scans, was divided into discrete blocks of 20 A-scans, yielding a total of 460 blocks (23×400/20=460) for training and LOOCV, which are visualized as individual data points in Figure 2a. While evaluating such homogeneous scans represents a relatively simplified task compared to highly heterogeneous transitional zones—which naturally leads to elevated baseline performance—this controlled environment is essential for establishing robust initial decision boundaries (Figure 2a).

Unlike binary approach, the training was structured as a multi-class problem (Normal tissue, Damaged Matter, and Tumor) using a One-vs-Rest (OvR) strategy. Prior to SVM fitting, the feature vectors were standardized to zero mean and unit variance using a standard scaler. The SVM was configured with a linear kernel to establish straightforward, interpretable decision boundaries between the tissue classes. We note that for such a limited dataset of 23 scans, employing a more complex, non-linear kernel would likely lead to overfitting. For our core objective, the absolute accuracy of the standalone SVM model is less critical than demonstrating how effectively the Human-in-the-Loop (HITL) approach compensates for the inevitable errors of a non-ideal algorithm during inference (note: no HITL intervention occurred during the training process; our focus is strictly on human–AI collaboration at the inference stage). To ensure robust performance evaluation, a leave-one-out cross-validation (LOOCV) procedure was utilized on the training dataset, where scaling and model fitting were strictly contained within each cross-validation fold.

Validation of this two-parameter model (OAC + RSC) demonstrated high discriminative capacity (Figure 2b). The multi-class LOOCV yielded Area Under the Curve (AUC) values of 0.966 for Normal tissue, 0.815 for Damaged Matter, and 0.928 for Tumor. For the critical binary task of separating Tumor from all other non-malignant tissues, the model achieved an AUC of 0.928. Bootstrapping (10,000 iterations) of the LOOCV predictions for this target cancer class demonstrated an accuracy of 84.1% [95% CI: 80.7–87.4], a sensitivity of 83.2% [95% CI: 78.1–88.0], and a specificity of 85.0% [95% CI: 80.3–89.4]. This automated A-scan classification served as the basis for the segmentation maps (Figure 1d) used in the third arm of the comparative study.

### 2.5. HITL Study Design

**Figure 3:**
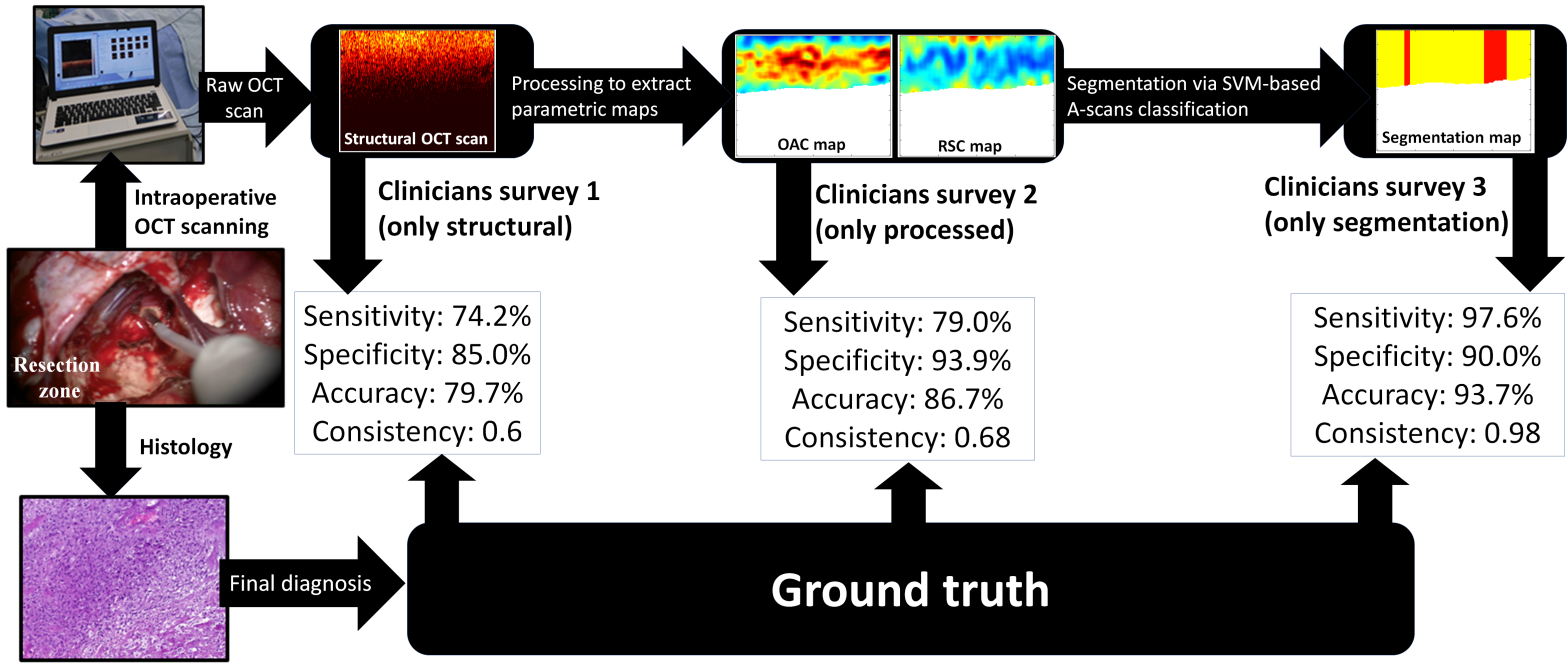
Schematic overview of the study design. Intraoperative OCT scanning acquiring a structural image. Resected tissue sample sent for histological analysis (gold standard). Processing of the structural scan using Equations (1) and (2) to calculate optical parameters. Generation of a segmentation map. Blinded randomized assessment at each of three steps by five neurosurgeons. Statistical comparison of the neurosurgeons’ assessments with histological results to calculate sensitivity, specificity, and consistency.

This was a single-center, retrospective study. It is important to emphasize that the image analysis was performed exclusively by these clinical neurosurgeons, rather than by OCT specialists. Thus, the study design is inherently translational in nature, specifically aimed at evaluating the practical capabilities of OCT as an optical biopsy method during brain glioma resection. The study design is presented in Figure 3. All scanned tissue samples were excised and submitted for histological examination. Following acquisition, the scans were processed, and an A-scan classification algorithm was applied to generate segmentation maps.

We defined visual criteria for all types of scans. The visual criteria for tumor structural scans included: deeper attenuation, a non-uniform horizontal attenuation line, and uneven signal intensity. All scan sets were randomized, anonymized, and presented to five neurosurgeons who had no prior experience with OCT. First, the specialists attended a brief lecture on how to analyze all scan types. Subsequently, they were asked to analyze each group separately.

We performed three equal groups of assessment: (1) Assess structural scan; (2) assess parametric maps; (3) assess segmentation map. Each group contained all 86 scans, presented in a unique random sequence.

Crucially, to reflect the clinical workflow and evaluate the genuine interpretability of each modality, neurosurgeons were required to make a forced choice between ‘White Matter’ and ‘Tumor’ (no ‘Uncertain’ option was provided). To assess the clarity of the visual information, we evaluated the inter-rater consistency (the degree of agreement among the five experts). A high consistency alongside high accuracy indicates a robust and easily interpretable imaging modality. The results were compared against the gold standard of histological examination.

### 2.6. Gemini 3.1 Pro as a Baseline Model

To rigorously evaluate the proposed HITL approach, we established a baseline using a state-of-the-art multimodal reasoning Large Language Model (LLM). Specifically, we utilized Gemini 3.1 Pro [20], leveraging its advanced spatial awareness, visual pattern recognition, and inherent complex reasoning capabilities. The model was queried directly through the Google AI Studio interface (https://aistudio.google.com/).

To facilitate in-context learning, we employed a visual grid prompting strategy. We designed a balanced 3 × 3 image grid layout. While modern models like Gemini 3.1 Pro are not strictly bound by the small native resolution limits of earlier vision encoders, the 3 × 3 dimension was specifically selected to prevent visual overloading and maintain the model’s attention on the fine morphological details and color boundaries of the tumor infiltration patterns. Furthermore, colorbars were cropped from the original segmented images to eliminate irrelevant visual noise.

Each grid consisted of exactly one test image placed in the top-left position (1,1) and eight contextual reference images. To strictly eliminate class imbalance during the in-context learning phase, the reference examples were perfectly distributed to include exactly four Class 1 (norm) and four Class 2 (tumor) samples, which were randomly shuffled across the remaining grid cells.

To overcome the “anchor bias” typically associated with hard categorical outputs, the prompt required the model to first explicitly identify the Ground Truth (GT) coordinates of the reference images to verify its spatial awareness, and then output a granular continuous tumor probability (from 0% to 100%) for the test image. Since Gemini 3.1 Pro is a reasoning model, we relied on its native thinking process rather than applying external step-by-step structures. The exact prompt provided to the model was as follows:

*[SYSTEM_ACTIVATION: NEURO_ONCOLOGY_EXPERT]*

***# System***

*You are an expert neuro-oncologist with advanced visual acuity, specializing in cancer cell infiltration analysis using segmented Brain OCT scans.*

***# Context***

*I am providing you with an image. The image is a 3 × 3 grid of segmented Brain OCT scans. Colorbars and axes have been removed to focus strictly on tissue morphology.*

*GRID COORDINATE SYSTEM: The grid uses a (row, column) format from (1,1) to (3,3). The ‘TEST IMAGE’ is always at position (1,1). The other 8 cells contain reference examples (exactly four ‘Class 1: Normal’ and four ‘Class 2: Tumor’).*

*CRITICAL DIAGNOSTIC RULES:*

*1. Tissue Zones: Green = Normal WM, Yellow = Damaged WM, Red = Tumor.*

*2. Segmentation Noise: The segmentation model produces false positives.*

*3. Neighborhood Context: If Red lines are rare and surrounded mostly by Green lines, they are highly likely False Positives. If Red lines are surrounded by Yellow lines, tumor probability increases. High frequency and dense clustering of Red lines indicate a very high probability of cancer.*

*4. Vertical Depth: True cancer (Red) lines typically extend deeper vertically towards the bottom boundary.*

***# Task***

*Perform an analysis to:*

*Step 1: Identify the exact coordinates (row,col) of all 4 ‘Class 1’ and all 4 ‘Class 2’ references, strictly excluding position (1,1).*

*Step 2: Analyze the frequency, neighborhood context, and depth of the Red lines strictly in the TEST IMAGE at (1,1).*

*Step 3: Compare the TEST IMAGE patterns to the reference examples you located.*

*Step 4: Conclude with a precise Tumor Probability specifically for the TEST IMAGE at (1,1).*

***# Output Format***

*Only output the final results in the following EXACT format:*

*Filename from (1,1) position: <Name.png>*

*Class 1 locations excluding position (1,1): (r,c), (r,c), (r,c), (r,c)*

*Class 2 locations excluding position (1,1): (r,c), (r,c), (r,c), (r,c)*

*Tumor Probability in grid position (1,1): <0 to 100>%*

This specific prompting architecture serves as a robust In-Context Learning mechanism, utilizing a perfectly balanced visual grid (exactly four Class 1 and four Class 2 reference samples) to mitigate any majority-class bias. Furthermore, by explicitly requiring the model to locate and output the Ground Truth (GT) coordinates, we not only verified its spatial visibility accuracy but also forced the network to anchor its visual attention, a step that inherently improves the model’s overall perception and reasoning capabilities. We note here that we did not perform prompt learning to tune the prompt for peak performance. Because the model operates in a reasoning mode, the generation temperature was set to 1.0 to maximize its complex analytical capabilities, allowing it to thoroughly evaluate nuanced visual heuristics before generating the final probability score. To ensure strict independence and prevent any potential data leakage between evaluations, each image grid was processed within a completely new and empty context window.

**Figure 4:**
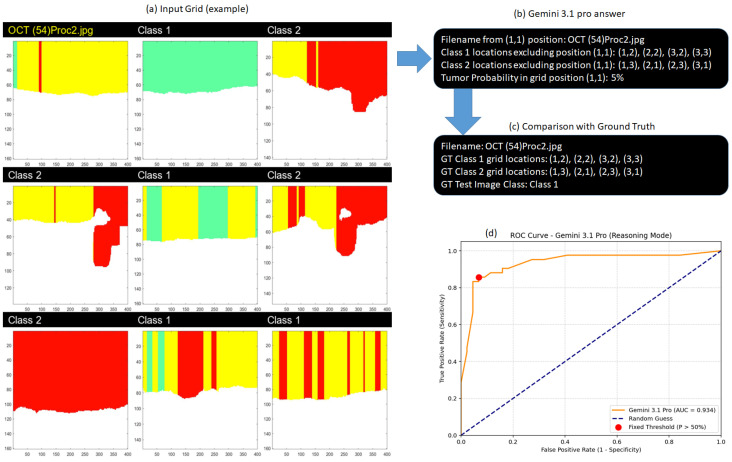
Workflow and evaluation of the Gemini 3.1 Pro baseline model in Reasoning Mode. (**a**) An example of the balanced 3×3 input grid provided to the model, containing the test image at the top-left position (1,1) alongside eight distributed reference scans providing contextual learning to the VLM. (**b**) The structured text output generated by the model, detailing the recognized grid coordinates of the reference classes and the predicted tumor probability for the test image. (**c**) The corresponding Ground Truth (GT) data used to automatically verify the model’s spatial awareness (GT visibility accuracy) and classification correctness. (**d**) The Receiver Operating Characteristic (ROC) curve derived from the continuous probability scores across the test dataset, achieving an AUC of 0.934. The red dot highlights the fixed binary classification threshold at P>50%.

By instructing the model to output a continuous probability rather than a binary label, we were able to construct a ROC curve to evaluate its diagnostic discriminative ability. We calculated the AUC. Rather than dynamically tuning an optimal operational threshold via statistical methods (such as Youden’s J statistic), we evaluated the binary classification performance using a fixed, intuitive threshold of P>50% (i.e., predicting a tumor if the output probability strictly exceeds 50%). This fixed cutoff was chosen to mirror a direct clinical decision-making process, allowing for a fair comparison against human experts who provide definitive binary assessments. The complete analytical workflow, including the input grid, the model’s structured response, the ground truth verification, and the final ROC curve, is summarized in Figure 4.

### 2.7. Statistical Analysis

To estimate the statistical uncertainty and comprehensively quantify the performance of both the human experts and the Gemini 3.1 Pro baseline model, we employed a non-parametric bootstrapping technique. This model-free approach allowed us to rigorously calculate the 95% confidence intervals for accuracy, sensitivity and specificity.

Due to the high computational cost and rate limits associated with querying the Gemini 3.1 Pro Vision-Language Model, as well as the practical impossibility of repeatedly surveying human experts thousands of times, the bootstrapping was not applied to the model executions or the surveying processes themselves. Instead, it was applied to the final paired arrays of ground truth labels and their corresponding predicted outputs (human diagnoses or AI probabilities).

Specifically, we generated 10,000 bootstrap iterations by randomly sampling the prediction pairs with replacement, strictly maintaining the original sample size (N=86) for each iteration. For the Gemini baseline, performance metrics were recalculated for each resampled set using the fixed P>50% operational threshold. For the human readers, the metrics were derived directly from their definitive binary assessments. This resampling process created an empirical distribution for each individual metric. The 95% CIs were subsequently derived by extracting the 2.5th and 97.5th percentiles from these respective distributions. All statistical analyses and plot generations were implemented using Python 3.12.12.

## 3. Results

### 3.1. Diagnostic Performance and Consistency

The diagnostic performance of the three OCT scan types was assessed by five neurosurgeons. Table 1 presents the confusion matrices for each scan type. Since a forced-choice paradigm was utilized, all 430 assessments (86 scans × 5 surgeons) are distributed among true/false positive/negative categories.

Based on these raw counts, Sensitivity, Specificity, Global Accuracy, and Inter-rater Consistency were calculated (Table 2). Inter-rater consistency serves as a quantitative measure of the method’s interpretability and the operators’ collective confidence.

The generated segmentation maps demonstrated superior overall performance. Crucially, the human inference on Segmentation maps established a definitive, statistically significant superiority over structural scans across sensitivity, global accuracy, and inter-rater consistency. They likewise significantly outperformed the SVM baseline in both sensitivity and accuracy, as well as human inference on Processed maps in sensitivity and consistency. Notably, while the Processed (Parametric) Maps improved Specificity and Global Accuracy compared to structural scans, they exhibited a relatively moderate inter-rater human inference consistency (0.68 [0.59–0.78]), reflecting the cognitive difficulty of interpreting continuous variable maps compared to the almost perfect and significantly higher consensus (0.98 [0.95–1.00]) achieved with segmentation maps. When compared to the advanced Gemini 3.1 Pro baseline, the human inference on Segmentation maps maintained a pronounced and significant edge in sensitivity (98% [92–100] vs. 86% [74–95]). Regarding global accuracy, while the human-in-the-loop approach on segmentation maps yielded a robust 94% [88–98], this improvement falls slightly short of achieving strict statistical significance over the SOTA Gemini 3.1 pro model (90% [83–95]) due to the overlap in their confidence intervals.

### 3.2. Cases Demonstrating HITL Effectiveness and Limitations

**Figure 5:**
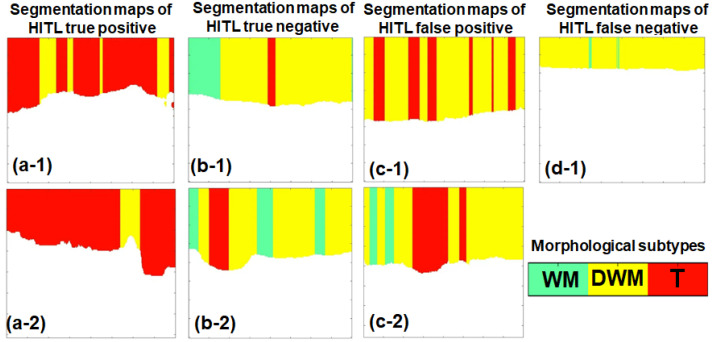
Illustrative examples of typical HITL classification outcomes and human–model interaction dynamics. (**a-1**,**a-2**) Two examples of HITL true positive: The algorithm correctly identifies tumor tissue (red A-scans), and the surgeon validates the morphological pattern, confirming the diagnosis. (**b-1**,**b-2**) Two examples of HITL true negative: The algorithm erroneously generates false-positive red stripes; however, the surgeon recognizes the biologically implausible pattern as artifactual noise, overrides the AI in 9 out of 13 cases (69%), and correctly classifies the scan as negative. (**c-1**,**c-2**) Two examples of HITL false positive: The algorithm generates a misleadingly plausible cluster of false-positive A-scans; the surgeon is fooled by the morphology and erroneously confirms the positive diagnosis (occurring in 4 out of 13 cases, or 31%). (**d-1**) The single example of HITL false negative: The algorithm fails entirely to detect the tumor, and the operator, lacking any visual warnings, concurs with the incorrect negative assessment (observed in only 1 case).

To illustrate the practical value, dynamics, and limitations of the Human-in-the-Loop approach, Figure 5 presents specific instances ranging from successful human–AI synergy to systemic failures.

#### 3.2.1. HITL True Positive (Human Confirms AI)

In this scenario (Figure 5a), the ML algorithm correctly detects tumor tissue, presenting contiguous red A-scans. The surgeon assesses the overall morphological pattern, agrees that the sequence aligns with biological expectations for tumor infiltration, and confidently confirms the positive diagnosis. This represents ideal, harmonious human–AI collaboration where both are correct.

#### 3.2.2. HITL True Negative (Human Overrides AI Error)

This scenario (Figure 5b) highlights the core operational benefit of the HITL framework. The algorithm erroneously detects tumor cells within healthy tissue (which occurred in 13 cases overall), producing artifactual red stripes (an algorithmic false positive). However, the human operator recognizes this non-anatomical, scattered distribution as noise. Relying on their domain knowledge of tissue topology, the surgeon rejects the AI’s suggestion in 9 of these cases and overrides the machine. Ultimately, the diagnosis is marked as negative, matching the ground truth. Here, the human effectively filters out the machine’s error, producing a HITL True Negative.

#### 3.2.3. HITL False Positive (Synergistic Error)

Here (Figure 5c), both the algorithm and the operator fail. The ML model produces a false positive, generating a cluster of red A-scans. Unfortunately, this specific combination morphologically mimics actual tumor infiltration. The surgeon is misled by this plausible presentation and erroneously confirms the positive diagnosis. The resulting HITL False Positive demonstrates the limit of human contextual filtering when algorithmic noise aligns too closely with biological expectations.

#### 3.2.4. HITL False Negative (Systemic Limitation)

In this scenario (Figure 5d), the algorithm completely misses the tumor, displaying no red A-scans (an algorithmic false negative). Lacking both algorithmic red flags and obvious visual structural cues, the surgeon agrees with the negative assessment, despite the ground truth being a tumor. This represents a complex case where the pathology is invisible in the current feature space to both the machine and the human. Notably, out of the 42 actual tumor scans evaluated, 41 were unanimously identified correctly (true positives) by the operators. This single case was the only one misclassified as a false negative, a direct consequence of the complete absence of algorithmic tumor indicators on the ML map.

## 4. Discussion

This study demonstrates that the diagnostic accuracy of intraoperative OCT is significantly influenced by the level of data processing presented to the surgeon. Our results establish a clear hierarchy of diagnostic performance, and importantly, reveal a critical “interpretability gap” in quantitative imaging when assessed through inter-rater consistency. We emphasize that in this analytical context, inter-rater consistency serves as a quantitative metric of method interpretability and collective operator confidence, rather than a standalone measure of clinical validity. The qualitative assessment of structural scans yielded the lowest overall diagnostic accuracy (80% [95% CI: 73–86%]) and consistency (0.60 [95% CI: 0.49–0.70]), as useful information is often masked by attenuation and speckle noise. The transition to quantitative processed maps (OAC and RSC) theoretically mitigates these issues by visualizing direct optical properties. Indeed, the forced-choice methodology revealed that the global accuracy of parametric maps increased to 87% [95% CI: 82–92%] and specificity to 94% [95% CI: 89–98%]. However, mentally mapping continuous variable colors to tissue states is cognitively demanding. This difficulty manifested as a moderate inter-rater consistency of 0.68 [95% CI: 0.59–0.78] (Table 2), indicating that without extensive training, parametric maps still introduce some ambiguity into the decision-making process.

The segmentation maps bridge this gap by inherently simplifying the visual landscape through a higher level of abstraction, which directly drives the maximized diagnostic consensus among operators (0.98 [95% CI: 0.95–1.00]). However, it is critical to acknowledge that high inter-rater agreement reflects consistency, not necessarily correctness; if the segmentation algorithm systematically produces large errors, it could theoretically bias all reviewers toward a shared incorrect diagnosis. Our results explicitly refute the presence of such blind algorithmic adherence. If the surgeons were merely passively reading and accepting the SVM’s segmented outputs, their diagnostic metrics would strictly mirror the standalone SVM baseline (Accuracy 84% [95% CI: 81–87%], Specificity 85% [95% CI: 80–89%]). Notably, the standalone SVM performance is significantly lower than even the human inference on continuous processed scans. Yet, human inference on the segmented scans achieved a superior, statistically significant accuracy of 94% [95% CI: 88–98%]. This significant diagnostic improvement over the SVM baseline definitively proves that operators are actively correcting the algorithm. Furthermore, the accompanying surge in consistency to 0.98 indicates that they are making these corrections highly unanimously.

This dynamic perfectly encapsulates the value of the HITL approach specifically at the inference stage. The human–model synergy is most impactful not in straightforward cases, but within the model’s “gray zones” of high uncertainty. For instance, the model may display a sparse number of potentially malignant cells that technically fall within the expected false-positive ratio for the scan. In these ambiguous zones, clinicians apply supplementary, unquantifiable domain knowledge that is entirely absent from the model’s feature space—such as recognizing realistic topological patterns of cancer cell infiltration versus the structural destruction of normal tissue. The operators act as an advanced contextual filter, effectively resolving the model’s uncertainty where it fails. Consequently, this fusion of human and machine at the inference stage significantly improves overall diagnostic performance, elevating it well above both the human-only and models-only baselines.

From a methodological standpoint, we deliberately chose a simple linear SVM classifier to maximize algorithmic transparency for this specific human-in-the-loop inference study. While modern deep learning approaches (such as CNNs) represent the current standard in medical image segmentation, their training requires significantly larger datasets than were available for this study. The classical ML approach remains the most viable option in our data-scarce regime. Importantly, the core premise of our study is not the absolute supremacy of the standalone ML model, which inevitably generates false-positive classifications at the individual A-scan level and exhibits “grey zones” of high decision uncertainty. Rather, our focus is on demonstrating that human operators can effectively correct these model errors during inference. The human–model fusion significantly improves overall performance above both the autonomous ML output and human-only assessments of raw scans. This was rigorously confirmed by benchmarking the HITL inference against the standalone SVM model and the state-of-the-art multimodal Vision-Language Model, Gemini 3.1 Pro. While advanced reasoning models like Gemini exhibit powerful contextual capabilities, the direct integration of human domain knowledge proved superior, firmly establishing the value of HITL over fully autonomous “models-only” pipelines. Our findings indicate that human intervention is most effective precisely in zones of high algorithmic uncertainty. In cases where the model visualizes a sparse number of potential cancer cells—which could easily fall within the false-positive ratio of the scan—clinicians are able to apply their supplementary, non-quantifiable domain knowledge. By evaluating the morphological plausibility of infiltration patterns relative to normal tissue architecture and destruction, the human operator acts as a critical post-processing filter. They successfully override algorithmic noise in 69% (9 out of 13) of false-positive cases because tissue topology and signal margins provide vital contextual cues that the algorithm currently lacks.

A potential source of systematic bias must be considered regarding the lack of prior OCT experience among all participating neurosurgeons. As the participants were novices to this specific modality, the observed advantage of segmentation maps may partially reflect a “learning or guidance effect”—simplifying data interpretation for first-time users—rather than the absolute diagnostic superiority of the method alone. There is an inherent risk that non-specialists might over-rely on the algorithm’s simplified output. However, our findings suggest that this risk is significantly mitigated by the operators’ underlying medical expertise. As demonstrated in Section 3.2.2 (Human Overrides AI Error), although they lacked OCT experience, the surgeons utilized their deep domain knowledge of neuroanatomy and tumor biology. By projecting their understanding of realistic tissue structure and impossible infiltration pathways onto the ML-generated spatial maps, they were able to perceptually evaluate the model’s uncertainty and successfully filter out biologically implausible false positives. Importantly, similar dynamics were observed in the prompt-guided reasoning of the Gemini 3.1 Pro benchmark. This VLM-based test, utilizing few-shot in-context learning, effectively simulates an OCT-inexperienced but contextually guided specialist who can successfully mitigate false-positive errors immediately following structured guidance. While this highlights the strong translational value of our approach for a broad clinical audience, it warrants further investigation. Future studies should include a comparison with OCT-experienced experts to determine how the baseline level of imaging expertise influences the dynamics of human–AI collaboration.

From a practical perspective, the proposed HITL framework demonstrates high translational relevance for real-time intraoperative integration. One of the key advantages of our approach is the substantial reduction in diagnostic time. The entire cycle of OCT scanning (approximately 3 s per volume) and segmentation map generation takes approximately 2 min, enabling prompt clinical decision-making during surgery without significantly increasing overall procedural duration. The system is designed to seamlessly fit into the standard glioma resection workflow, allowing surgeons to view real-time OCT scans alongside segmented images on a monitor. Furthermore, in the surgical environment, the clinician continuously recalibrates their risk tolerance for known ML errors in real-time, adjusting the balance of diagnostic risk based on the immediate surgical context, such as the functional eloquence of the brain area being resected.

Despite these promising results, several limitations of the current study must be explicitly acknowledged. First, the study relies on a relatively small, single-center dataset (27 patients, 86 scans) that pools multiple distinct glioma grades into a single heterogeneous cohort. This oversimplifies the highly variable structural and textural characteristics of different tumor subtypes. Second, the reliance on prior work from our group for the training dataset (23 B-scans) introduces a risk of circular validation. The lack of an independent, external validation cohort increases the risk of overfitting and may lead to overly optimistic performance claims. Third, the current binary classification framework (Tumor vs. Non-tumor) fails to account for “damaged white matter” (used as a proxy for tumor risks in our approach) as a distinct and clinically adverse pathology, thereby limiting the overall clinical relevance of the evaluations. Finally, the sequential presentation of the three imaging modalities to the raters may have introduced a learning or order effect, potentially biasing interpretations over time despite the randomization of scans within each block. Future studies must employ a fully randomized or counterbalanced design and transition to a multi-class assessment framework.

To overcome these limitations and move toward broad clinical implementation, independent external multi-center validation is strictly required. A larger, multi-center cohort is necessary to stratify results by specific glioma grades and properly capture the complex biological context of tumor invasion. Recognizing that data scarcity is the primary bottleneck in this domain, we aim to ignite collaborative, multi-centric research. To this end, upon acceptance of this manuscript, we will make our completely anonymized dataset of intraoperative in vivo OCT scans publicly available on Zenodo. We invite the global community to utilize these data, combine them with their own datasets, and actively participate in refining and validating advanced human-in-the-loop inference or agentic frameworks for OCT-based neurooncology.

## 5. Conclusions

This study confirms that a “Human-in-the-Loop” (HITL) workflow, integrating machine learning-based segmentation with human contextual analysis, delivers the highest diagnostic accuracy and reliability for intraoperative OCT in glioma surgery. We identified that, while quantitative parametric maps contain rich diagnostic information and improve global accuracy (87% [95% CI: 82–92%]), they suffer from limited interpretability, leading to moderate inter-rater consistency of 0.68 [95% CI: 0.59–0.78]. The segmentation maps bridge this gap, simplifying the visual landscape and maximizing diagnostic consensus among operators to 0.98 [95% CI: 0.95–1.00], while achieving the highest overall diagnostic performance (Accuracy 94% [95% CI: 88–98%] and Sensitivity 98% [95% CI: 92–100%]).

By directly benchmarking this HITL inference against fully autonomous “models-only” pipelines, we demonstrated its clear superiority. The HITL approach significantly outperformed the standalone SVM classifier (Accuracy 84% [95% CI: 81–87%]; Sensitivity 83% [95% CI: 78–88%]). Furthermore, it surpassed the contextually prompted SOTA multimodal reasoning model, Gemini 3.1 Pro, during inference on SVM-based segmentation maps (Accuracy 90% [95% CI: 83–95%]; Sensitivity 86% [95% CI: 74–95%]). While the human-driven sensitivity maintained a definitive, statistically significant edge over the Gemini model, the accuracy improvement fell just slightly short of undisputed statistical significance due to overlapping confidence intervals.

Crucially, despite the inevitable presence of algorithmic false positives that autonomous models alone could not resolve, operators successfully apply their contextual domain knowledge of tumor invasion patterns and topological priors to recognize and filter out 69% (9 out of 13) of this algorithmic noise. This HITL synergy not only improves overall diagnostic performance by optimally utilizing human contextual intelligence over purely algorithmic reasoning, but also ensures that the ultimate decision-making responsibility remains safely with the clinician.

The clinical relevance of these findings is underpinned by a uniquely valuable dataset and an inherently translational study design. All images were acquired directly during intraoperative in vivo OCT imaging, followed by the precise excision of the region of interest for subsequent pathomorphological examination. Furthermore, it is important to emphasize that the analysis of the OCT images was performed exclusively by clinical neurosurgeons, rather than by OCT specialists. Thus, the study design directly evaluates the practical capabilities of OCT as an intraoperative optical biopsy method during brain glioma resection.

Importantly, this work is devoted to the HITL approach solely at the inference stage, rather than during model learning. This means that there was no specific human involvement in the SVM model training beyond providing the initial histologically validated training dataset. Instead, all human intervention occurred exclusively during inference—whether the operators were analyzing structural scans, processed parametric maps, or evaluating the final segmentation maps generated by the SVM model. By positioning the AI as an integrated collaborative tool rather than an autonomous decision-maker, the HITL framework empowers surgeons to confidently override inevitable algorithmic errors using their broader clinical domain knowledge. This synergistic approach ensures that human experts remain firmly in control of the final diagnostic inference, while simultaneously highlighting the specific boundaries where future AI feature engineering is required.

## Figures and Tables

**Table 1:** Confusion matrices for the assessment of OCT scan types (86 scans × 5 neurosurgeons leads to n = 430 assessments per type).

Assessment Type	True Positives	True Negatives	False Positives	False Negatives
Structural scans	156	187	33	54
Processed scans	158	202	13	42
Segmentation maps	205	198	22	5

**Table 2:** Diagnostic performance metrics of the assessed OCT scan types. Values are presented as percentages with 95% confidence intervals in brackets, calculated via scan-level bootstrapping (10,000 iterations).

Assessment Type	Sensitivity (%)	Specificity (%)	Global Accuracy (%)	Consistency
Structural scans (Human inference)	74 [63–84]	85 [78–91]	80 [73–86]	0.60 [0.49–0.70]
Processed scans (Human inference)	79 [72–88]	94 [89–98]	87 [82–92]	0.68 [0.59–0.78]
Segmentation maps (Human inference)	98 [92–100]	90 [81–97]	94 [88–98]	0.98 [0.95–1.00]
SVM (baseline)	83 [78–88]	85 [80–89]	84 [81–87]	Not applicable
Gemini 3.1 pro (baseline)	86 [74–95]	93 [85–100]	90 [83–95]	Not applicable

## Data Availability

The data presented in this study are available in this article or Supplementary Material. All scan examples used in this study are available in the Supplementary Material. To support reviewer-requested reproducibility and open science, and to facilitate independent multi-center validation, the intraoperative in vivo OCT scans used in this study are attached to this paper under a Conditional Non-Exclusive License. To the best of our knowledge, this dataset represents the first publicly available intraoperative in vivo OCT scans of human brain gliomas globally. Under this Conditional Non-Exclusive License, the use of these materials is permitted subject to prior notification of the authors and on the condition that this article is appropriately cited. The data used in this retrospective study were originally acquired during RSF project No. 23-75-10068; however, that project did not provide financial support for the specific research presented in this paper. The code that converts OCT scans into physics-based maps (OAC and RSC) can be found at https://github.com/SynthOCTChallenge/SynthOCT_Baseline (accessed on 28 April 2026) (Part3_Processor.py) and on the OpticElastograph LLC cloud-based platform: https://accounts.opticelastograph.com (accessed on 28 April 2026) (requires registration). These solutions were developed under RSF project No. 25-12-20032, which solely funded this research.

## Supplementary Materials

The following supporting information can be downloaded at: https://www.mdpi.com/article/10.3390/medsci14020263/s1.
